# Systematic reviews and meta-analyses on major depressive disorder: a bibliometric perspective

**DOI:** 10.3389/fpsyt.2023.1136125

**Published:** 2023-04-26

**Authors:** Pan Chen, Yuan Feng, Xiao-Hong Li, Jia-Xin Li, Yue-Ying Wang, Wan-Ying Zheng, Zhaohui Su, Teris Cheung, Gabor S. Ungvari, Chee H. Ng, Sha Sha, Yu-Tao Xiang

**Affiliations:** ^1^Unit of Psychiatry, Department of Public Health and Medicinal Administration, Faculty of Health Sciences, Institute of Translational Medicine, University of Macau, Macau, Macao SAR, China; ^2^Centre for Cognitive and Brain Sciences, University of Macau, Macau, Macao SAR, China; ^3^Beijing Key Laboratory of Mental Disorders, The National Clinical Research Center for Mental Disorders, Advanced Innovation Center for Human Brain Protection, Beijing Anding Hospital, Capital Medical University, Beijing, China; ^4^Beijing Huilongguan Hospital, Huilongguan Clinical Medical School, Peking University, Beijing, China; ^5^School of Public Health, Southeast University, Nanjing, China; ^6^School of Nursing, The Hong Kong Polytechnic University, Hong Kong, Hong Kong SAR, China; ^7^Section of Psychiatry, University of Notre Dame Australia, Fremantle, WA, Australia; ^8^Division of Psychiatry, School of Medicine, University of Western Australia, Perth, WA, Australia; ^9^Department of Psychiatry, The Melbourne Clinic and St Vincent’s Hospital, University of Melbourne, Richmond, VC, Australia

**Keywords:** depression, bibliometric analysis, evidence-based medicine research, systematic review, meta-analysis

## Abstract

**Background:**

There is a vast amount of evidence-based medicine research on the major depressive disorder (MDD) available in the literature, however, no studies on the overall performance, productivity and impact of such research have been published to date. This study explored and mapped the research outputs of MDD-related systematic reviews and meta-analyses (SR/MA) from a bibliometric perspective.

**Methods:**

Relevant data were retrieved with search terms on MDD, systematic review and meta-analysis.

**Results:**

A total of 4,870 papers with 365,402 citations published from 1983 to 2022 were included in the analysis. The publication output has grown steadily over time with the most publications originating from the USA (1,020; 20.94%), the UK (516; 10.60%) and China (448; 9.20%). The research collaborations between countries were most frequent between the USA and UK (266; 5.46%). Journal of Affective Disorders (379; 7.78%) was the most productive journal, while Cuijpers P was the most productive author (121; 2.48%), and University of Toronto (569; 11.78%) was the most productive institution. The top 10 most cited articles on MDD-related SR/MA had citations ranging from 1,806 to 3,448. The high-frequency keywords were mainly clustered into four themes, including psychiatric comorbidities, clinical trials, treatment, and brain stimulation in MDD.

**Conclusion:**

The rapid increase in the number of SR/MA of MDD in recent years highlights the importance of this research field. Psychiatric comorbidities, clinical interventions, and treatment of MDD have been identified as hot topics, while biological mechanisms in MDD are likely to be an emerging research priority.

## 1. Introduction

Major depressive disorder (MDD) is one of the most prevalent mental disorders. According to the criteria of the Diagnostic and Statistical Manual of Mental Disorders-5, MDD is defined as having one or more major depressive episodes without lifetime mania and hypomania, and is characterized by at least five out of nine clusters of symptoms (e.g., depressed mood, change in weight or appetite, insomnia or hypersomnia, observed psychomotor retardation or agitation, loss of energy, feeling of worthlessness or guilt, impaired concentration or indecisiveness, and suicidal ideation or attempt) that is present nearly every day within the past 2 weeks and causes significant distress or impairment (1). Due to its high prevalence, disease burden and health economic cost (2), MDD has gained increasing research attention. Previous bibliometric research studies on MDD (3, 4), have covered a wide range of different aspects such as epidemiology, etiology, symptomatology, diagnosis, treatment and prognosis, in which evidence-based medicine (EBM) approach is widely applied (5). For example, a systematic review (SR) and meta-analysis (MA) of 20 studies examined the global prevalence of MDD in older population (13.3%; 95% CI: 8.4–20.3%) (6). Another MA of 36 randomized clinical trials (RCTs) found that anti-inflammatory drugs could improve the effects of antidepressant treatment (7).

Evidence-based medicine refers to the integration of the best available research evidence combined with clinical expertise, knowledge and patients’ values and choices (8, 9). Originally focused on critical appraisal and development of systematic reviews and clinical practice guidelines, EBM has become a key guiding principle for the investigation of clinical outcomes (10). According to the Oxford Centre for Evidence-Based Medicine, SR and MA are viewed as evidence with the highest quality level in EBM research (11). Thus, to certain extent, SR/MA can contribute to the development of EBM in a certain field (12, 13). SR can determine a defined clinical question and use explicit methods to perform a comprehensive literature search with strict inclusion and exclusion criteria and assessment of the included studies (8). In contrast, MA is essentially a type of SR that can statistically combine the data from eligible primary studies to produce a single pooled estimate with minimal heterogeneity (14). Thus, MA can generate a large sample size that a single study cannot achieve due to the limitations of resources, time, or other factors. Additionally, MA can resolve potentially conflicting findings for the same topic from research (10). Both evidence-based methods give a comprehensive and precise review of a given topic providing guidance for further exploration. To date, there has been an extensive number of SR/MA in the MDD field, such as the prevalence of MDD in different groups (e.g., adolescents, older adults, comorbidities) (6, 15), medication treatment for MDD (e.g., statins, lithium) (16, 17) and biomarkers in patients with MDD (18, 19). However, no published study to date has systematically evaluated the overall performance, productivity and impact of such research on MDD-related SR/MA.

Bibliometric analysis is increasingly used to provide a macroscopic overview of the research trend of a particular topic and examine the knowledge structure of relevant publications at the literature level (20). It consists of performance analysis and science mapping, which quantitatively and visually present the scientific output and the collaborations between each component (20). Furthermore, a key advantage of bibliometric analysis is citation analysis. As SR/MA usually have high citations (4, 21), the bibliometric analysis could explore the degree of impact of MDD-related SR/MA. Previous bibliometric analyses have examined EBM in various fields, such as general medicine and health sciences (22), dentistry (23), and tuberculosis (24), which have provided an overview of the development structure of the respective fields. However, no bibliometric studies on MDD-related EBM have yet been published.

Given the large number of MDD-related SR/MA and their critical role in clinical practice guidelines development, the analysis of research trends and gaps could guide future directions of research and set priorities for key research and clinical stakeholders. Therefore, this study aimed to explore and map the publication outputs of MDD-related SR/MA from a perspective of bibliometric analysis, including the trend of publications (numbers and citations), sources of publications (e.g., country, authorship, journal), collaborative networks between each component, research hotspots and future frontiers of MDD related SR/MA.

## 2. Materials and methods

### 2.1. Data source and search strategy

**FIGURE 1:**
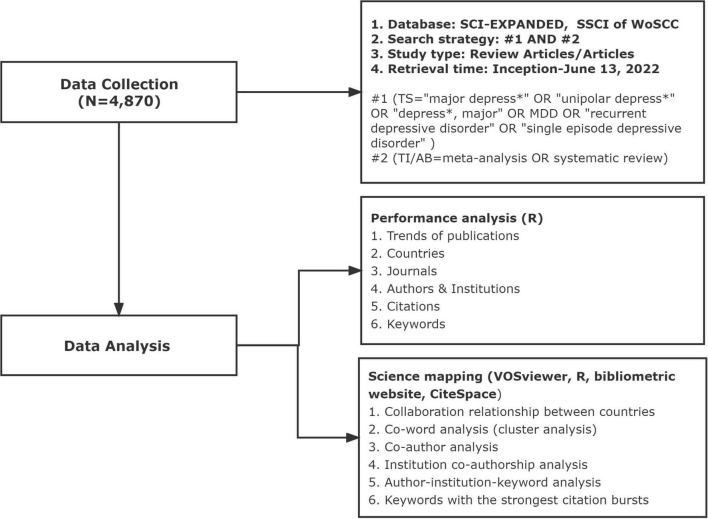
Flowchart of data collection and study design.

In bibliometric analysis, the Web of Science is the most widely used database with several advantages including having a broad array of fields, earlier data inception, and a powerful citation network (25). Data were retrieved from the Science Citation Index Expanded and Social Sciences Citation Index in the Web of Science Core Collection from their inception dates to 13 June 2022, with the following search terms: “(TS = “major depress*” or “unipolar depress*” or “depress*, major” or MDD or “recurrent depressive disorder” or “single episode depressive disorder”) and (TI/AB = meta-analysis or systematic review).” Publication types included reviews and original articles. The data was extracted from publications and then exported as the format of “Plain text file” or “Tab-delimited file,” and were recorded as “Full record and cited references.” The flow chart of data collection is shown in Figure 1.

### 2.2. Data analysis

The method of synthetic analysis (21) was adopted to overview the research trend of EBM research on MDD both quantitatively and qualitatively using bibliometric tools, including R software (version 4.2.0), VOSviewer software (version 1.6.17) and CiteSpace software (6.1.R2) (26).

*Bibliometrix* package in R software is a comprehensive tool to conduct the performance analysis and science mapping of the publications (27). The function used in this study covered the key information of publications (numbers and citations), source of publications (countries and journals), authorship of publications (institutions and authors), and collaborative relationships between countries (visualized by the *Bibliometric* website).^1^ Additionally, the unique Sankey diagram was used to visualize three fields (authors, institutions, and keywords) to reveal their relationships (28). The h-index was applied to evaluate the quality of publications, and the 2021 impact factor was used to evaluate the quality of journals (29). Moreover, the *mgcv* package in R software was adopted to conduct a generalized additive model to predict the trend and expected numbers of publications in EBM research on MDD over the years (21, 30).

*VOSviewer* software is a commonly used visualization tool for science mapping (31). It adopts the visualization for cluster analysis (32) and displays three types of visualization maps: network, overlay, and density. This study generated three sets of network maps and corresponding overlay maps according to the types of nodes (authors, institutions, and keywords) and explored the collaborative relationships between authors, institutions, and popular topics in EBM research on MDD. A node in the maps represents an author, an institution, or a keyword. For author and institution maps, the size of the node reflects the number of publications (NP) published by an author or an institution. For keywords maps, the size of nodes reflects occurrences of keywords and the nodes with different colors are clustered as different topics. The thickness of edges linked by nodes represents the strength of the associations between nodes. The total link strength (TLS) refers to the total number of co-occurrences of an individual node with other nodes; the higher the number of co-occurrences, the stronger association is between nodes (33). Further, the overlay maps provide a view of the changes of nodes over time based on the associations, and the colors are ranked from blue to yellow by the average publication year (APY) of articles.

CiteSpace software is a scientific knowledge mapping tool (34). The function of keywords burst detection was used in this study. This could reflect the evolution of research hotspots over time and predict the future priorities in a certain field. If keywords continued to be frequently cited until 2022, they were likely to represent future research priorities (35). In addition, the references with the strongest citation bursts were also analyzed to explore the most cited articles in recent years.

## 3. Results

### 3.1. Distribution of annual publications and citations

**FIGURE 2:**
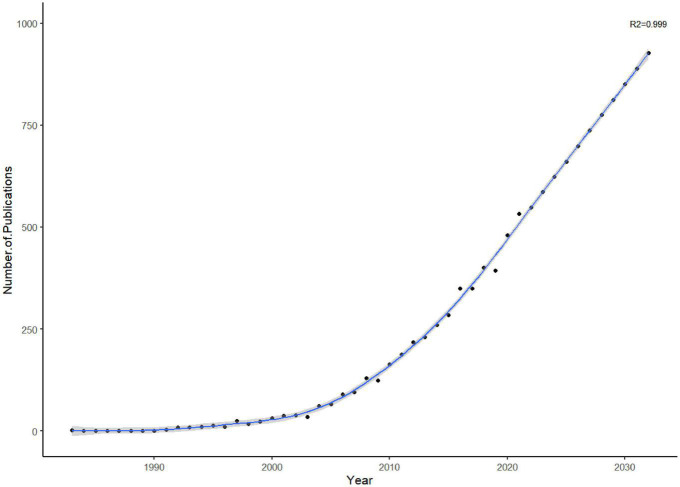
Trend of publications on MDD-related systematic review/meta-analysis (SR/MA).

Altogether, 4,870 papers with 365,402 citations published from 1983 to 2022 were included. Figure 2 shows the fitting curve of annual publications with a steadily growing trend of publications on MDD-related SR/MA, indicating increasing research attention to this area. The formula of the fitting curve was “Formula = *Number of Publications*∼ *s(Year).”* Supplementary Table 1 presents in detail the numbers of annual publications and average citations per year from 1983 to 2022.

### 3.2. Countries

**FIGURE 3:**
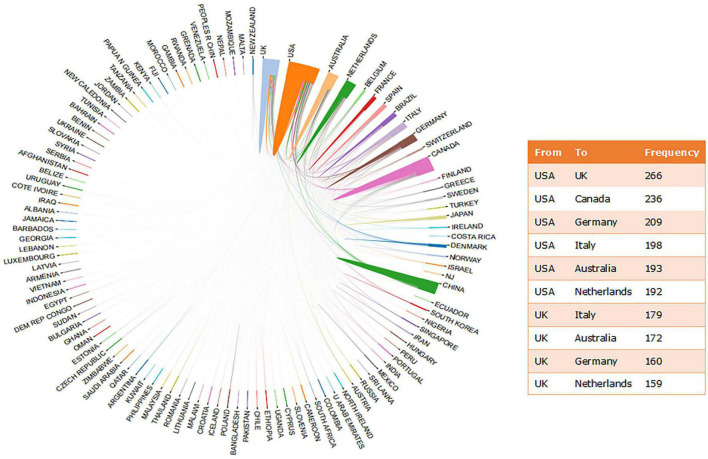
Collaborative research between countries on MDD-related SR/MA.

There were 104 countries involved in conducting MDD-related SR/MA. Supplementary Figure 1 shows the distribution of publications on MDD-related SR/MA by countries. The USA (5,760), UK (2,809), Canada (2,570), China (1,967), Australia (1,839), Germany (1,733), Netherlands (1,617), Italy (1,044), Spain (717) and Brazil (688) were the top 10 countries with the most publications. Eight of them are high-income countries according to the World Bank’s criteria (36). Figure 3 shows the collaborative network between countries. The research collaborations were most frequent between the USA and UK (266; 5.46%), followed by between the USA and Canada (236; 4.85%), and between USA and Germany (209).

If only corresponding authors were counted, then 65 countries were involved in the research on MDD-related SR/MA. Table 1 shows the distribution of the top 10 countries of the corresponding authors. The USA, UK, and China were ranked as the top three most productive countries. Corresponding authors in the USA had published 1,020 publications (20.94%) with 118,090 citations. A single-country publication (SCP) refers to an article in which all authors worked in the same country, while a multiple-country publication (MCP) refers to an article with at least two authors from different countries (37). The higher ratio of MCP, the stronger the multinational collaborations between countries (37). Thus, in the top 10 countries, Italy had the strongest international collaborations (MCP ratio = 0.624), while the USA had the strongest national collaborations (MCP ratio = 0.265).

**TABLE 1:** Top 10 most productive countries of corresponding authors on MDD-related SR/MA.

SCR	Country	Income level	Articles	Percent	TC	AAC	SCP	MCP	MCP_ratio
1	USA	High	1,020	20.94%	118,090	116	750	270	0.265
2	UK	High	516	10.60%	51,910	101	322	194	0.376
3	China	Upper middle	448	9.20%	12,386	28	306	142	0.317
4	Canada	High	441	9.06%	28,910	66	255	186	0.422
5	Germany	High	330	6.78%	18,996	58	229	101	0.306
6	Australia	High	312	6.41%	26,898	86	174	138	0.442
7	Netherlands	High	306	6.28%	30,579	100	146	160	0.523
8	Italy	High	242	4.97%	13,911	57	91	151	0.624
9	Brazil	Upper middle	148	3.04%	7,751	52	68	80	0.541
10	Denmark	High	142	2.92%	10,981	77	80	62	0.437

SCR, standard competition ranking; TC, total citations; AAC, average article citations; SCP, single country publications; MCP, multiple country publications.

### 3.3. Journals

The 4,870 included papers were published in 893 journals. Supplementary Table 2 shows the top 10 journals with the most publications. Journal of Affective Disorders (379; 7.78%), Journal of Clinical Psychiatry (112; 2.30%), and Psychological Medicine (96; 1.97%) were the top three most productive journals that published MDD-related SR/MA. Eight of 10 journals are in the category of Psychiatry/Psychology. One of the remaining two is the Cochrane Database of Systematic Reviews, which is the leading journal for systematic reviews in Health Care, while the remaining one is Neuroscience and Biobehavioral Reviews in Behavioral Sciences and Neurosciences. The 2021 impact factor of the top 10 journals ranged from 5.250 to 13.437. Based on the Journal Citation Reports, 80% of the 10 journals were classified as Q1. Supplementary Figure 2 shows the growth trend of publications for the top 10 journals. Of note, the Journal of Affective Disorders had a sharp increase in the number of publications after 2010.

### 3.4. Authors and institutions

A total of 19,364 authors from 4,831 institutions were involved in conducting MDD-related SR/MA. Table 2 shows the top 10 most active authors and their respective institutions. Cuijpers P from Vrije University Amsterdam contributed to the most publications (121; 2.48%) with a total of 15,901 citations, followed by Stubbs B from King’s College London (63; 1.29%), and Cipriani A from the University of Oxford (62; 1.27%). Cuijpers P also has the highest h-index value (38) among the top 10 authors. University of Toronto (569; 11.78%) was the most productive institution, followed by King’s College London (485; 10.04%) and Vrije Universiteit Amsterdam (365; 7.56%).

**TABLE 2:** Top 10 most active authors contributing to MDD-related SR/MA.

SCR	Author (*N* = 19,364)	H-index	TC	NP	SCR	Institution (*N* = 4,831)	NP	Percent
1	Cuijpers P (Vrije University Amsterdam)	60	15,901	121	1	University of Toronto	569	11.78%
2	Stubbs B (Kings College London)	34	6,647	63	2	King’s College London	485	10.04%
3	Cipriani A (University of Oxford)	29	5,520	62	3	Vrije Universiteit Amsterdam	365	7.56%
4	Mcintyre RS (University of Toronto)	32	3,305	59	4	McGill University	353	7.31%
5	Furukawa TA (Kyoto University)	25	4,515	58	5	The University of Melbourne	292	6.04%
6	Carvalho AF (Deakin University)	32	4,297	57	6	The University of Edinburgh	211	4.37%
7	Barbui C (University of Verona)	25	3,041	42	7	University of Calgary	209	4.33%
8	Vieta E (University of Barcelona)	24	2,554	42	8	University of Oxford	203	4.20%
9	Boomsma DI (Vrije University Amsterdam)	28	7,115	41	9	Harvard University	189	3.91%
10	Brunoni AR (University of São Paulo)	27	2,949	41	10	Harvard Medical School	172	3.56%

SCR, standard competition ranking; TC, total citations; NP, number of publications.

Supplementary Figure 3A presents the co-authorship networks, which included 55 nodes (authors) with a TLS of 1,178, with each author having published at least 15 articles. Stubbs B shared the strongest collaboration with others (TLS: 163), followed by Carvalho AF (TLS: 152) and Solmi M (TLS: 127). In addition, the two thickest lines were between Stubbs B and Vancampfort D (green color), and between Cipriani A and Fornaro M (purple color), indicating that the two pairs of authors had the strongest collaborations. As shown in Supplementary Figure 3B, Stubbs B (APY: 2017.96; NP: 60; TLS: 163), Mcintyre RS (APY: 2017.84; NP: 60; TLS: 87) and Carvalho AF (APY: 2017.94; NP: 52; TLS: 152) had published the most publications on MDD-related SR/MA and had the most collaborations with others in recent years (yellow color).

Supplementary Figure 3C presents the collaborations between institutions. They included 68 nodes (institutions) with a TLS of 4,250 with each institution having published at least 35 articles. King’s College London had the strongest collaboration with other institutions (TLS: 568), followed by the University of Toronto (TLS: 556), and Vrije Universiteit Amsterdam (TLS: 315). Supplementary Figure 3D shows that Harvard Medical School (APY: 2019.30; NP: 100; TLS: 178), South London and Maudsley NHS Foundation Trust (APY: 2018.25; NP: 81; TLS: 279), and Deakin University (APY: 2018.29; NP: 70; TLS: 192) had published more publications on MDD-related SR/MA and had more collaborations with others in recent years (yellow color).

Further, Supplementary Figure 4 shows the collaborations between authors and institutions on certain topics. For example, Mcintyre RS published the most articles with the keywords of “depression” through the University of Toronto.

### 3.5. Most cited articles

Supplementary Table 2 shows the top 10 most cited articles on MDD related SR/MA with the citations ranging from 1,806 to 3,448. The topics mainly focused on epidemiology/disease burden (39–42), comorbidities (39, 43), screening tools (44), prevention (45) and biological mechanisms (46–48). The article entitled “*Global, regional, and national incidence, prevalence, and years lived with disability for 301 acute and chronic diseases and injuries in 188 countries, 1990–2013: a systematic analysis for the Global Burden of Disease Study 2013*” from the Lancet had the most citations (*N* = 3,448) (41).

Supplementary Figure 5 presents the top 10 references with the strongest citation bursts in the past 5 years; of them, the citation burst for two articles published in *The Lancet* had rapidly increased, entitled “Comparative efficacy and acceptability of 21 antidepressant drugs for the acute treatment of adults with major depressive disorder: a systematic review and network meta-analysis” (49) (2020–2022) and “Depression” (2020–2022) (50), indicating that these topics (e.g., the efficacy of antidepressants; epidemiology, diagnosis, pathology, and management of MDD) are likely to be of future interest and priorities in the field of MDD-related SR/MA.

### 3.6. Keywords

#### 3.6.1. Keywords co-occurrence networks

**FIGURE 4:**
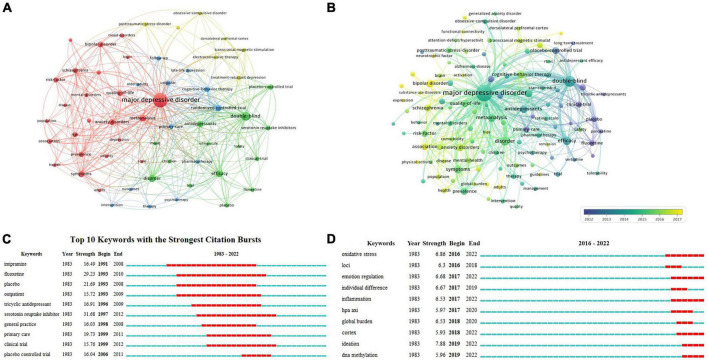
Analysis of the research hotspots on MDD-related SR/MA [**(A)** network visualization map of keywords co-occurrence; **(B)** overlay visualization map of keywords; **(C)** top 10 keywords with the strongest citation bursts; **(D)** keywords with the strongest bursts from 2016 to 2022].

Of the 8,784 keywords extracted from the 4,870 articles, the keywords that occurred more than 100 times were analyzed with 21,134 TLS (Figure 4A). “Major depressive disorder” (2,601), “double-blind” (897), and “disorder” (552) were the most frequent keywords. Figure 4A shows the 57 keywords in 4 clusters. Cluster 1 (red color) refers to the psychiatric comorbidities of MDD, such as “bipolar disorder,” “anxiety disorders,” “schizophrenia,” “prevalence,” and “risk factors.” Cluster 2 (green color) refers to clinical trials on MDD such as “double-blind,” “placebo-controlled trials,” and “clinical trials.” Cluster 3 refers to the treatment for MDD (blue color) such as “cognitive behavior therapy,” “follow up,” “pharmacotherapy,” and “psychotherapy.” Cluster 4 refers to the brain stimulation treatments of MDD (yellow color) such as “transcranial magnetic stimulation,” “prefrontal cortex,” and “electroconvulsive-therapy.”

Figure 4B shows the time change of these keywords. Keywords colored in yellow indicate that they have been used in recent years such as “bipolar disorder” (APY: 2016.89) and “genome-wide association” (APY: 2016.79). Keywords related to drugs or trials appeared earlier, with an APY before 2013, such as “fluoxetine” (APY: 2009.66), and “clinical trial” (APY: 2011.93).

To examine the transition of topics over time, the study period was split into three stages [1983–2002 (*n* = 226), 2003–2012 (*n* = 1,167), and 2013–2022 (*n* = 3,477)], and the analysis on publication topics was repeated within each stage. The results showed that the distribution of topics was similar between the stages (Supplementary Figure 6), indicating that the findings on the topics of overall publications had been constant over time.

#### 3.6.2. Keywords with the strongest citation bursts

Figure 4C presents the top 10 keywords that had the strongest citation bursts with a minimum duration of 3 years during the period from 1983 to 2022. The keywords “imipramine” (1991–2008), “fluoxetine” (1993–2010), and “outpatient” (1993–2009) received the longest attention in the earlier period. Figure 4D shows the burst keywords in the past 5 years. “Oxidative stress” (2016–2022), “emotion regulation” (2017–2022), “inflammation” (2017–2022), “cortex” (2018–2022), “ideation” (2019–2022), and “DNA methylation” (2019–2022) had the strongest bursts until 2022, indicating that they are emerging future research priorities in MDD-related SR/MA.

## 4. Discussion

To the best of our knowledge, this was the first bibliometric analysis to provide an overview of EBM research in the MDD field. The publications on MDD-related SR/MA showed an increasing trend during the past decades, which is consistent with the previous bibliometric analysis in the area of psychology (51), and are associated with the increasing trends of publications in the MDD field overall (4, 52). MDD is a public health priority with a high prevalence and has gained increasing attention in medical research (53). Moreover, the notable increase in the number of publications on MDD-related SR/MA highlights the importance and the need to promote the development of EBM in the MDD field.

The included papers were published by researchers distributed globally, which is in line with the global challenge of MDD and the worldwide attention to EBM in MDD. This is also consistent with the large overlap found between the overall scientific publications on depressive disorders and SR/MA on MDD (52). Nationally, scientific outputs are usually related to the economic status of a country (54). As shown in this study, most of the top 10 productive countries are ranked as high-income countries that have a greater capacity to provide research funds for the MDD field (55). The USA and the UK were among the most productive countries that also had the strongest collaboration in conducting MDD-related SR/MA. With a long tradition in psychology, the USA and the UK have been among the leading countries in the development of psychology and psychiatry (4). Similar to previous studies, they had the most publication number across various research areas such as psychiatric comorbidities of COVID-19 (56), schizophrenia (57), and depression or anxiety associated with coronary heart disease (35).

The analysis of journals can be informative to authors when submitting an article to a journal for publication (4). All the top 10 journals with MDD-related SR/MA were at the higher-ranking level in the Psychiatry/Psychology categories, which are important platforms for the exchange of research findings on MDD-related SR/MA. In this study, the Journal of Affective Disorder published the most MDD-related SR/MA, accounting for 7.78% of total publications, followed by the Journal of Clinical Psychiatry (2.30%), and Psychological Medicine (1.97%). Further, the publication number in the top 10 journals had a rapidly increasing trend over time, indicating that EBM on MDD had attracted increasing attention, for example, the Journal of Affective Disorders showed a sharp growth since 2010.

The co-authorship analysis could identify the most productive research teams and the collaborations between teams in a certain field. Scholars might exchange ideas and share academic resources through collaborations, which is necessary to promote the development of science and human health (25). In addition, a time map could be used to provide an overview of the dynamic development of co-authorship (32). We found that Cuijpers P from Vrije University Amsterdam, who conducted MDD-related SR/MA since an earlier period, had the most research impact with the most publications and total citations. In contrast, Stubbs B from King’s College London had the strongest collaboration with other groups, and published more publications on MDD-related SR/MA in recent years. The top three active institutions included the University of Toronto, King’s College London, and Vrije Universiteit Amsterdam, all of which had both the highest total number of publications and the strongest collaborations with others, indicating that they have been long established in this research area. In recent years, Harvard Medical School, South London and Maudsley NHS Foundation Trust, and Deakin University had produced more publications on MDD-related SR/MA, which suggests that they are emerging collaborators for scholars in future research. This study also examined the relationships between authors and institutions on certain topics, which could further provide directions to identify collaborators (4).

Highly cited articles could reveal important evidence in scientific research, and also give a historical perspective of scientific progress in a specific field (58). Further, the citations of an article could reflect its influence on the scientific community (59). In this study, compared to other topics, the SR/MA on MDD-related epidemiology had more citations; for instance, the highest cited article was published in Lancet by Global Burden of Disease Study 2013 Collaborators, which estimated the epidemiology and disease burden of 301 acute and chronic diseases and injuries in 188 countries between 1990 and 2013 (41). This SR showed that the prevalence of MDD changed by 53.4% (95% CI: 49.0–58%) from 1990 to 2013 and had become the second leading cause of years lived with disability globally by 2013. Other epidemiology reviews with high citations included the prevalence of comorbid depression in adults with diabetes (39); obesity and depression (43); perinatal depression (40) and genetic epidemiology of MDD (47). The findings focused on the relationship between MDD and its comorbidities, which could improve the awareness of early detection, prevention and treatment for those at risk (43). In addition, one SR published in JAMA summarized the effects of different interventions against suicide and proposed multiple prevention strategies. The study found that physician education in recognition and treatment of MDD, restriction of lethal methods, and gatekeeper education were the most effective interventions (45). As suicide is the most serious consequence of MDD (60), this high-cited SR would be critical to establishing recommendations for the future suicide prevention program. The results of the analysis on bursts of cited references indicated that the treatment, epidemiology, diagnosis, pathology, and management of MDD would remain hot topics of MDD-related SR/MA.

Keywords co-occurrence analysis could reveal hotspots that are most studied and give a perspective on the distribution of topics within a particular academic discipline (25). Our study found four themes in MDD-related SR/MA studies. Psychiatric comorbidities were the most studied theme in the keywords network analysis such as anxiety, schizophrenia and bipolar disorder. Previous studies found a strong and frequent bidirectional correlation between MDD and other psychiatric problems (38). For example, a study showed that 46–67% of patients with MDD also met the criteria for anxiety disorder (61–63) and 63% of anxiety disorder patients met the criteria for current MDD (63, 64). Mental disorders were often viewed as traumatic experiences that could trigger a depressive episode, and these disorders could share similar pathogenic mechanisms and risk factors (38, 65). Further, comparative analyses between different psychiatric disorders were often conducted due to certain overlapping symptoms, such as MDD and bipolar disorder (66, 67). Thus, the findings of SR/MA on MDD and other psychiatric disorders could increase the understanding of the relationship between these disorders.

Apart from “Major depressive disorder,” the term “double-blind” was a frequently used keyword, which is related to RCTs, inferring that the RCT design predominated in MDD-related SR/MA studies. According to the evidence level, the systematic review of RCTs has the highest quality level of evidence to establish causal associations in clinical research (68). Compared with other study designs, the use of randomization in RCTs could minimize variation and bias in group characteristics that might affect outcomes, thus providing strong evidence in terms of the effect of the intervention on outcomes (68). Moreover, the time analysis of keywords showed that studies on clinical trials of antidepressants were conducted earlier to develop strong evidence for effective treatments for MDD in the earlier stage.

The treatment of MDD is a high priority (69). The other two themes in the keywords network analysis were associated with the treatment of MDD, involving psychotherapy, pharmacotherapy and physical therapy; of these, cognitive behavior therapy (CBT) and electroconvulsive therapy (ECT) were commonly used in practice (70, 71). MDD is associated with cognitive impairment in multiple domains such as attention, memory, and executive functions, and the impairment could persist even in the remission of MDD (72, 73), which could partly explain the widespread use of CBT for MDD (74). In addition, ECT is an effective short-term treatment for depression, especially for treatment-resistant depression (70). Evidence showed that adjunctive use of MDD treatments was more efficient than monotherapy in improving mood symptoms such as the use of CBT or ECT plus antidepressants (69, 75). However, it was also noted that the treatment rate of MDD was still low across clinical settings globally (76).

Additionally, biological mechanisms of MDD might be future potential research priorities, which could guide further exploration to enhance the effectiveness of targeted MDD interventions. In recent years, increasing evidence showed that epigenetic modifications might play an important role in the pathogenesis of MDD (77). For example, DNA methylation might be a potential link between environmental factors and the occurrence of depression; a review showed that BDNF and NR3C1 gene methylation levels were associated with depression, but the relationship between SLC6A4 and depression was found to be contradictory.

There were several limitations in this study. First, following previous studies and relevant guideline recommendations for bibliometric analysis (20, 25, 35), publications included in this study were searched only with the Web of Science, which is the largest biomedical database and the most commonly used database for bibliometric analyses. Second, publications in non-English languages could not be included. Finally, some of the most cited articles published in the past may have later show to have limited benefit while some recent publications with low total citations may be neglected in the analyses.

In summary, the recent increase in the number of SR/MA publications highlights the importance of the MDD field. Of note, psychiatric comorbidities, clinical interventions, and treatments of MDD have been identified as hot topics in MDD-related SR/MA, while biological mechanisms in MDD are likely to be an emerging future research priority.

## Data availability statement

The original contributions presented in this study are included in this article/Supplementary material, further inquiries can be directed to Y-TX, xyutly@gmail.com.

## Author contributions

PC, YF, X-HL, SS, and Y-TX: study design. PC, YF, J-XL, Y-YW, W-YZ, ZS, TC, and GU: data collection, analysis, and interpretation. PC and Y-TX: drafting of the manuscript. CN: critical revision of the manuscript. All authors approved the final version for publication.

## Footnotes

1
https://bibliometric.com/
