# Supplementary material for: Systematic reviews and meta-analyses on major depressive disorder: a bibliometric perspective
Source: Front Psychiatry. 2023 Apr 26;14:1136125. doi: 10.3389/fpsyt.2023.1136125 (PMC10169641; doi:10.3389/fpsyt.2023.1136125)
Supplement: Supplementary file 1 [file Data_Sheet_1.docx]

**Supplementary Materials**

Figure S1. World map for the distribution of publications on MDD related SR/MA by country

Figure S2. The top 10 sources of publications on MDD related SR/MA

Figure S3. Map of collaboration networks of co-authorship analysis (A. Network visualization map of institutions; B. Network visualization map of authors; C. Overlay visualization map of institutions; D. Overlay visualization map of authors)

Figure S4. Three-Fields Plot of the keywords analysis (author-institution-keyword)

Figure S5. Top 10 references with the strongest citation bursts from 2016 to 2022

Figure S6. Analysis of the research hotspots on MDD related SR/MA in different stages (A. Network visualization map of keywords co-occurrence during 1983 and 2001; B. Network visualization map of keywords co-occurrence during 2002 and 2012; C. Network visualization map of keywords co-occurrence during 2013 and 2022)

Table S1. Number of annual publications on MDD related SR/MA

| **Year** | **NP** | **Percent** | **TC/Y** |
| --- | --- | --- | --- |
| 1983 | 1 | 0.02% | 2.49 |
| 1991 | 3 | 0.06% | 5.06 |
| 1992 | 9 | 0.18% | 6.45 |
| 1993 | 9 | 0.18% | 7.15 |
| 1994 | 10 | 0.21% | 5.87 |
| 1995 | 13 | 0.27% | 3.80 |
| 1996 | 10 | 0.21% | 1.88 |
| 1997 | 24 | 0.49% | 7.00 |
| 1998 | 17 | 0.35% | 3.36 |
| 1999 | 23 | 0.47% | 8.42 |
| 2000 | 31 | 0.64% | 8.60 |
| 2001 | 37 | 0.76% | 10.58 |
| 2002 | 39 | 0.80% | 9.45 |
| 2003 | 34 | 0.70% | 10.46 |
| 2004 | 61 | 1.25% | 9.99 |
| 2005 | 66 | 1.36% | 11.35 |
| 2006 | 89 | 1.83% | 12.13 |
| 2007 | 95 | 1.95% | 8.48 |
| 2008 | 130 | 2.67% | 9.78 |
| 2009 | 123 | 2.53% | 12.86 |
| 2010 | 164 | 3.37% | 13.31 |
| 2011 | 187 | 3.84% | 9.32 |
| 2012 | 218 | 4.48% | 10.57 |
| 2013 | 230 | 4.72% | 13.11 |
| 2014 | 260 | 5.34% | 10.38 |
| 2015 | 284 | 5.83% | 12.66 |
| 2016 | 349 | 7.17% | 10.08 |
| 2017 | 350 | 7.19% | 12.88 |
| 2018 | 401 | 8.23% | 10.49 |
| 2019 | 394 | 8.09% | 11.22 |
| 2020 | 480 | 9.86% | 7.80 |
| 2021 | 533 | 10.94% | 5.56 |
| 2022* | 196 | 4.02% | - |

Notes*: Italics: Predicted numbers of publications; * from January 1, 2022 to June 13, 2022*

Table S2. Top 10 productive journals on MDD related SR/MA

| **Sources** | **NP** | **Percent** | **IF (2021)** | **Category (JCR)** |
| --- | --- | --- | --- | --- |
| Journal of Affective Disorders | 379 | 7.78% | 6.533 | Clinical Neurology (Q1);  Psychiatry (Q1) |
| Journal of Clinical Psychiatry | 112 | 2.30% | 5.906 | Psychiatry (Q1);  Psychology, Clinical (Q1) |
| Psychological Medicine | 96 | 1.97% | 10.592 | Psychology, Clinical (Q1);  Psychology (Q1);  Psychiatry (Q1) |
| Neuroscience and Biobehavioral Reviews | 83 | 1.70% | 9.052 | Behavioral Sciences (Q1);  Neurosciences (Q1) |
| Journal of Psychiatric Research | 82 | 1.68% | 5.250 | Psychiatry (Q2) |
| Cochrane Database of Systematic Reviews | 79 | 1.62% | 12.008 | Medicine, General & Internal (Q1) |
| Molecular Psychiatry | 73 | 1.50% | 13.437 | Psychiatry (Q1);  Neurosciences (Q1);  Biochemistry & Molecular Biolog (Q1) |
| Acta Psychiatrica Scandinavica | 70 | 1.44% | 7.734 | Psychiatry (Q1) |
| Frontiers in Psychiatry | 70 | 1.44% | 5.435 | Psychiatry (Q2) |
| British Journal of Psychiatry | 66 | 1.36% | 10.671 | Psychiatry (Q1) |

Notes: IF: impact factor; JCR: Journal Citation Reports

Table S3. Top 10 most cited articles on MDD related SR/MA

| **SCR** | **Author & Year** | **Title** | **Journal (IF-2021)** | **TC** | **TC/Y** |
| --- | --- | --- | --- | --- | --- |
| 1 | Vos T, 2015 | Global, regional, and national incidence, prevalence, and years lived with disability for 301 acute and chronic diseases and injuries in 188 countries, 1990–2013: a systematic analysis for the Global Burden of Disease Study 2013 | Lancet (202.731) | 3,448 | 431 |
| 2 | Dowlati Y, 2010 | A Meta-Analysis of Cytokines in Major Depression | Biological Psychiatry (12.810) | 2,781 | 214 |
| 3 | Anderson RJ, 2001 | The Prevalence of Comorbid Depression in Adults with Diabetes: A meta-analysis | Diabetes Care (17.152) | 2,539 | 115 |
| 4 | Luppino FS, 2010 | Overweight, Obesity, and Depression: A Systematic Review and Meta-analysis of Longitudinal Studies | Archives of General Psychiatry (JAMA psychiatry) (25.911) | 2,227 | 171 |
| 5 | Wittchen HU, 2011 | The size and burden of mental disorders and other disorders of the brain in Europe 2010 | European Neuropsychopharmacology (5.415) | 2,133 | 178 |
| 6 | Kroenke K, 2010 | The Patient Health Questionnaire Somatic, Anxiety, and Depressive Symptom Scales: a systematic review | General Hospital Psychiatry (7.587) | 1,958 | 151 |
| 7 | Gavin NI, 2005 | Perinatal Depression: A Systematic Review of Prevalence and Incidence | Obstetrics and Gynecology (7.661) | 1,925 | 107 |
| 8 | Sullivan PF, 2000 | Genetic Epidemiology of Major Depression: Review and Meta-Analysis | American Journal of Psychiatry (19.242) | 1,901 | 83 |
| 9 | Mann JJ, 2005 | Suicide Prevention Strategies: A Systematic Review | JAMA-Journal of The American Medical Association (157.335) | 1,836 | 102 |
| 10 | Howren MB, 1997 | Associations of Depression With C-Reactive Protein, IL-1, and IL-6: A Meta-Analysis | Psychosomatic Medicine (3.864) | 1,806 | 129 |

Notes: TC/Y:Total Citations/Year; IF: impact factor; JCR: Journal Citation Reports


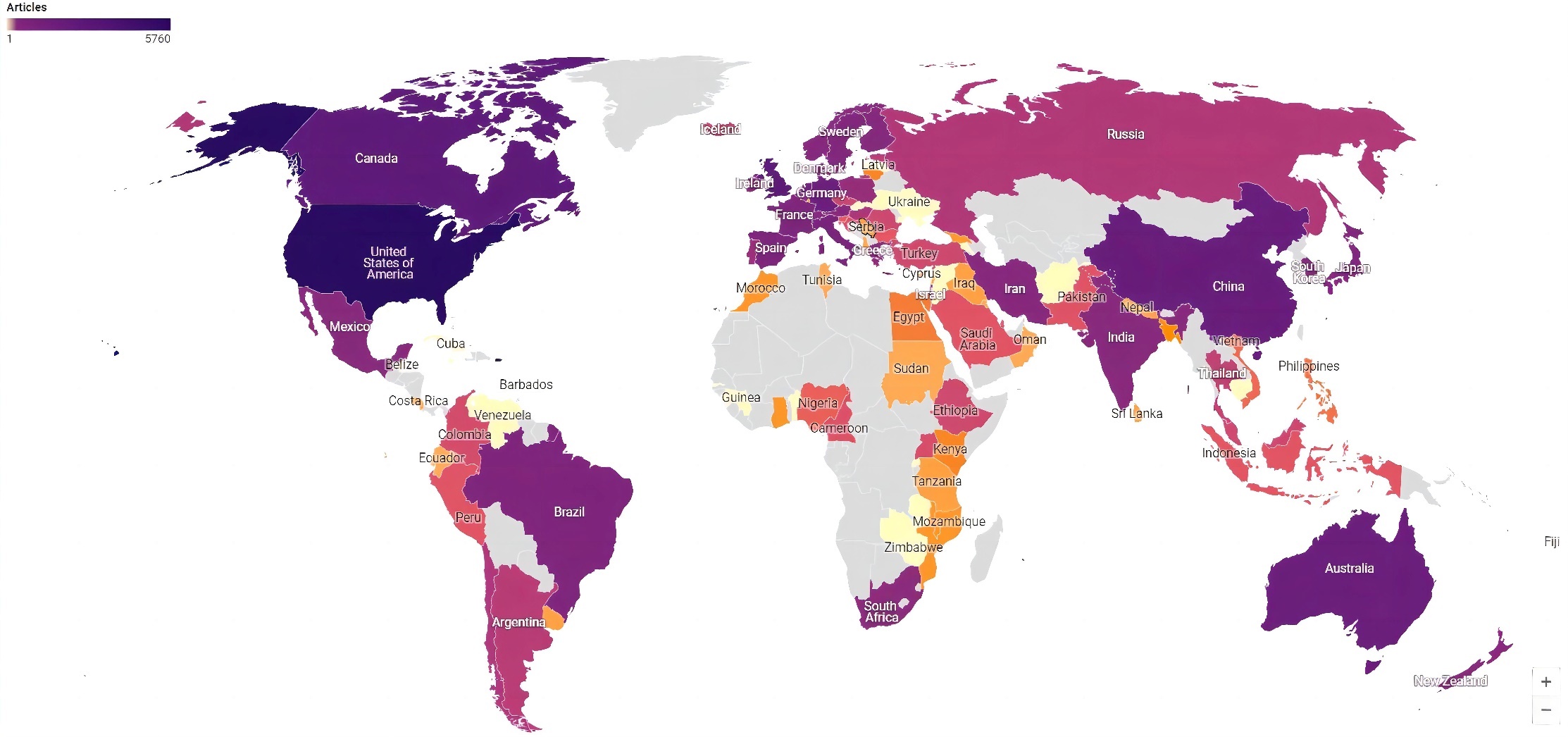


Figure S1. World map for the distribution of publications on MDD related SR/MA by country


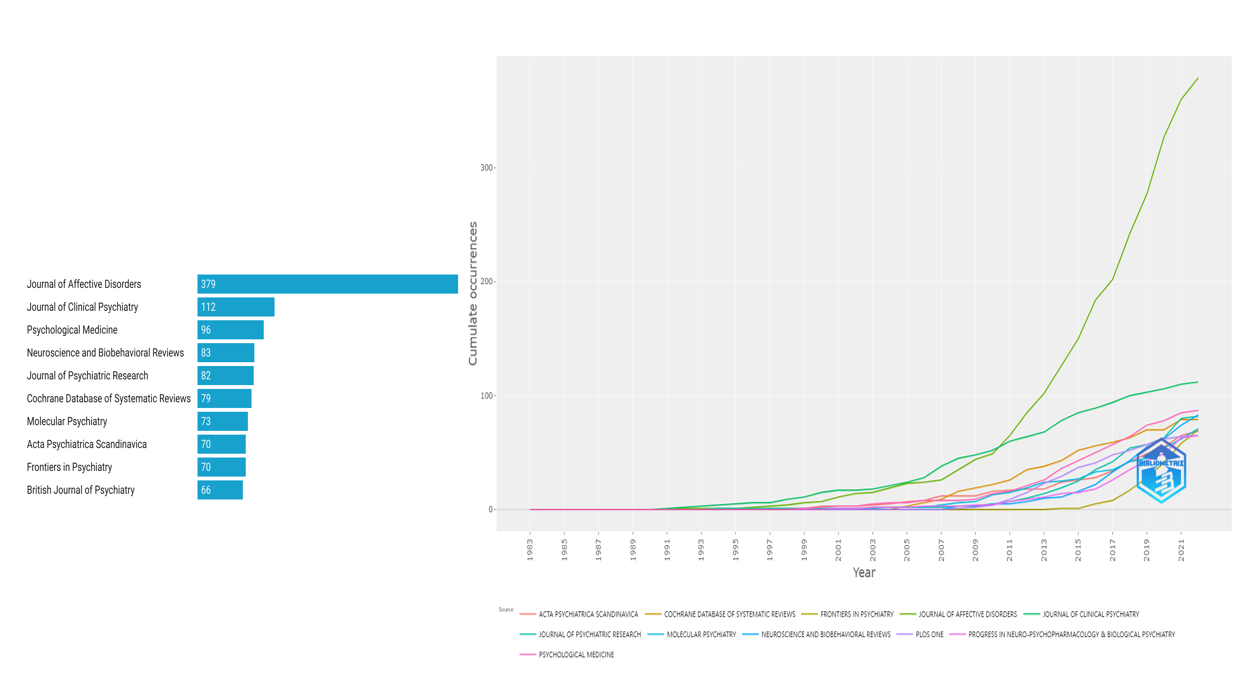


Figure S2. The top 10 sources of publications on MDD related SR/MA


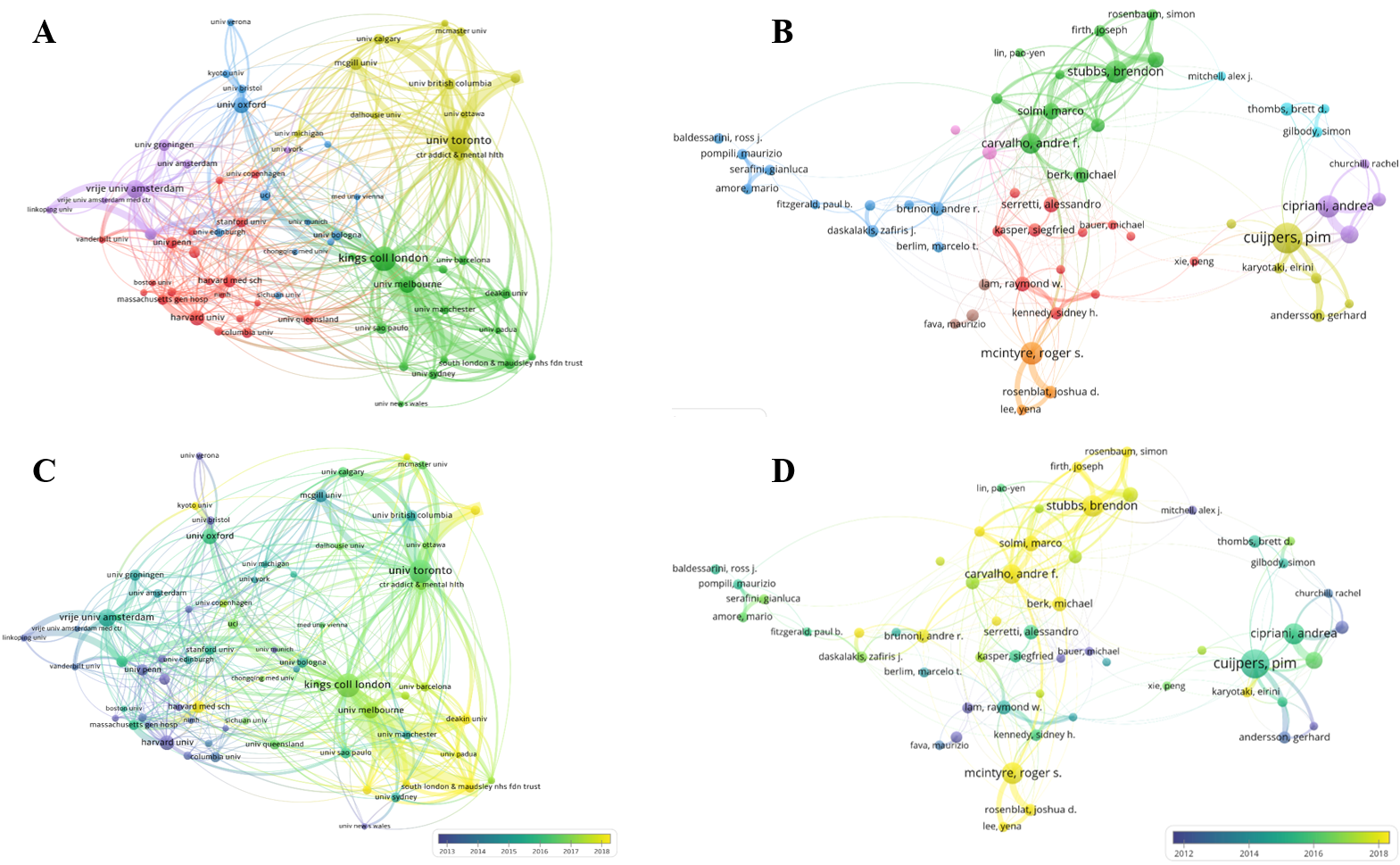


Figure S3. Map of collaboration networks of co-authorship analysis (A. Network visualization map of institutions; B. Network visualization map of authors; C. Overlay visualization map of institutions; D. Overlay visualization map of authors)


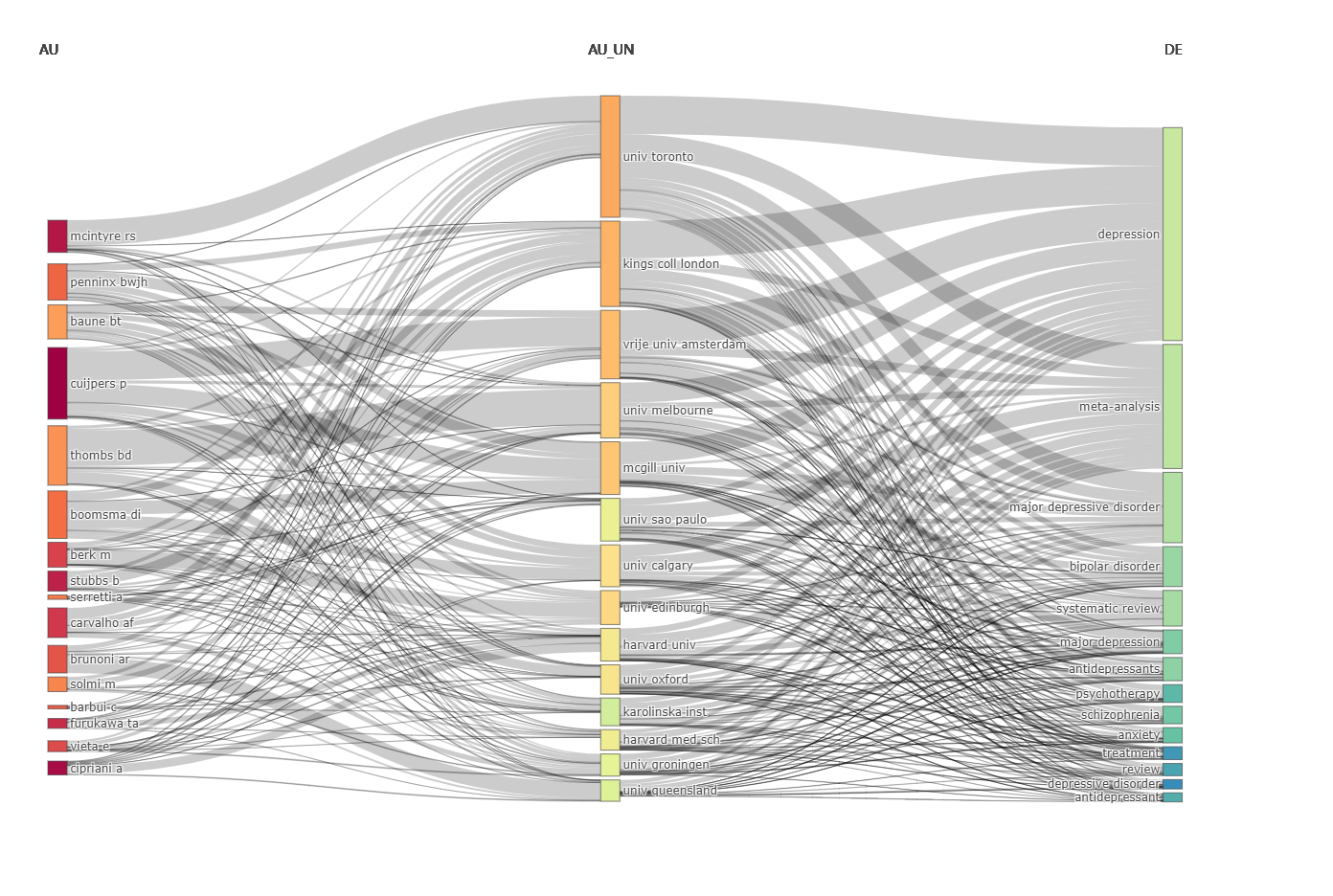


Figure S4. Three-Fields Plot of the keywords analysis (author-institution-keyword)


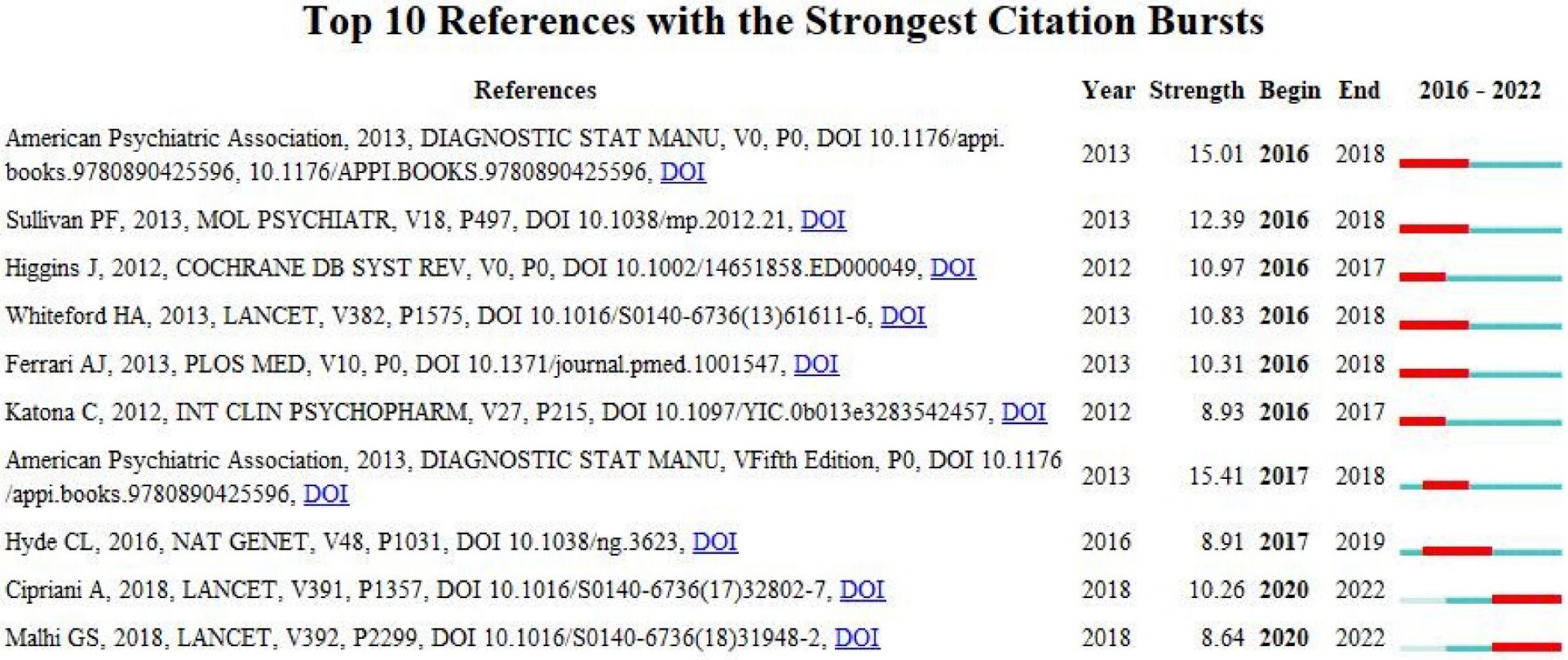


Figure S5. Top 10 references with the strongest citation bursts from 2016 to 2022


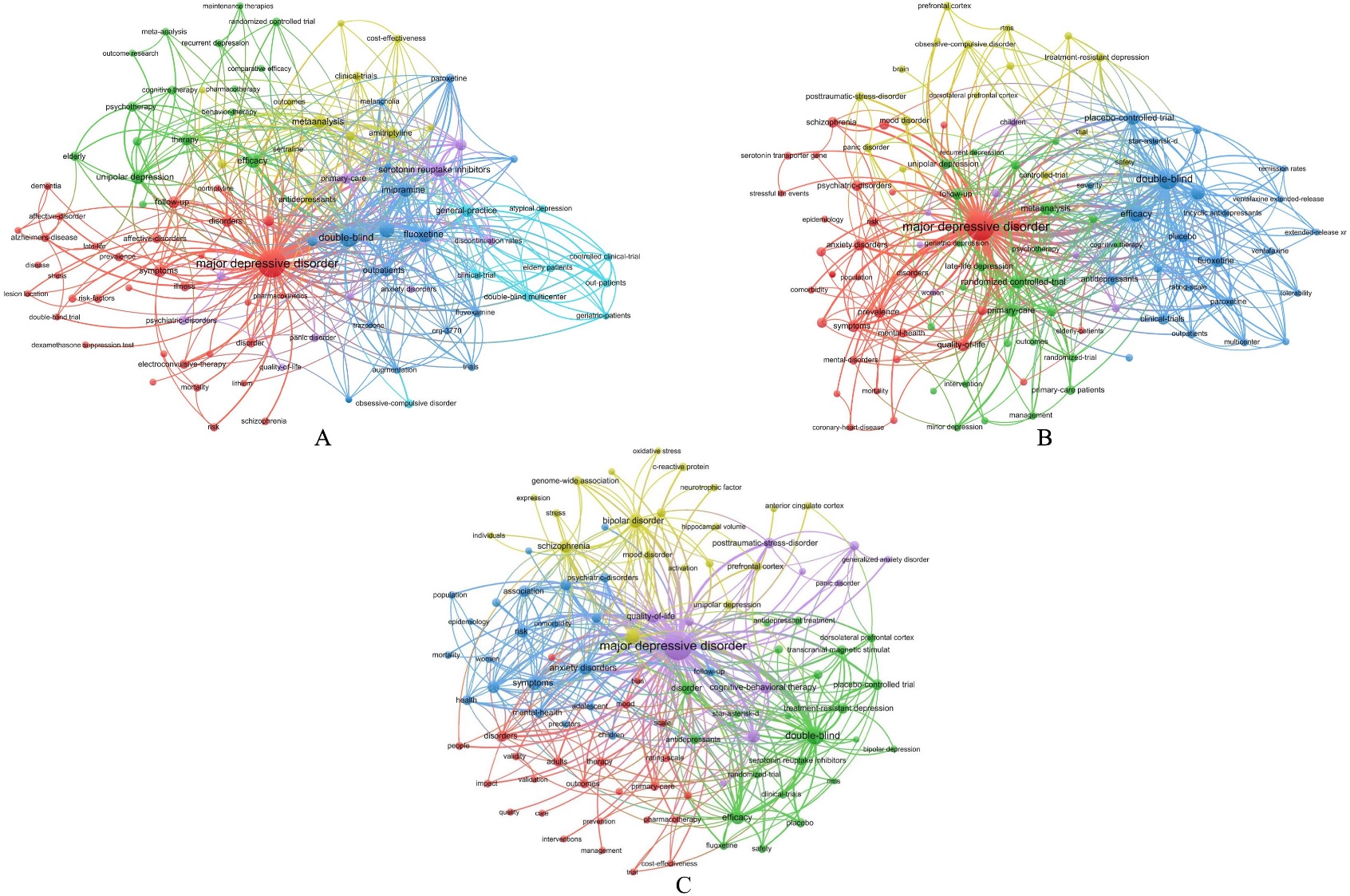


Figure S6. Analysis of the research hotspots on MDD related SR/MA in different stages (A. Network visualization map of keywords co-occurrence during 1983 and 2001; B. Network visualization map of keywords co-occurrence during 2002 and 2012; C. Network visualization map of keywords co-occurrence during 2013 and 2022)
